# Improvement of Chitosan Films Properties by Blending with Cellulose, Honey and Curcumin

**DOI:** 10.3390/polym15122587

**Published:** 2023-06-06

**Authors:** Noha G. Madian, Basant A. El-Ashmanty, Hadeel K. Abdel-Rahim

**Affiliations:** Biophysics Department, Faculty of Science, Cairo University, Giza 12613, Egypt; bassant.ahmed71997@gmail.com (B.A.E.-A.); hadeelkamal2812@gmail.com (H.K.A.-R.)

**Keywords:** chitosan, curcumin, antibacterial effect, crystallinity, close packing

## Abstract

Chitosan is a natural biopolymer that can be used in biomedical applications, tissue engineering, and wound dressing because of its biodegradability, biocompatibility, and antibacterial activity. The blending of chitosan films with natural biomaterials such as cellulose, honey, and curcumin was studied at different concentrations in order to improve their physical properties. Fourier transform infrared (FTIR) spectroscopy, mechanical tensile properties, X-ray diffraction (XRD), antibacterial effects, and scanning electron microscopy (SEM) were studied for all blended films. The XRD, FTIR, and mechanical results showed that films blended with curcumin were more rigid and compatible and had higher antibacterial effects than other blended films. In addition, XRD and SEM showed that blending chitosan films with curcumin decreases the crystallinity of the chitosan matrix compared to cellulose and honey blending films due to increased intermolecular hydrogen bonding, which reduces the close packing of the CS matrix.

## 1. Introduction

Improvement of the wound-healing process is one of the most important aspects of regenerative medicine. Different materials have been used to provide effective dressings for wounds. Chitosan (CS) is a biopolymer derived from chitin, which is the second natural polysaccharide after cellulose. Chitosan is a linear polysaccharide composed of randomly distributed -(14)-linked D-glucosamine and N-acetyl-D-glucosamine units that can be prepared by deacetylation of chitin. The hydroxyl group and amino group are functional groups of chitosan according to the properties of electron donors [1]. Chitosan is biocompatible, biodegradable, has antibacterial behavior, hemostatic characteristics, is inexpensive, is nontoxic, and can accelerate the wound healing process. All of these distinctive properties make it an attractive material for the treatment of wounds [2,3,4,5]. Consequently, it can be considered one of the most widely used dressing materials [5,6,7].

One of the drawbacks of CS film is its poor barrier and mechanical properties due to its sensitivity to environmental conditions, making it unsuitable for many applications. The incorporation of materials into CS thin films is considered an effective way to enhance their poor mechanical properties [8,9].

Blending and chemical modifications of CS by other polymers will change its mechanical properties [10]. Blending chitosan with natural materials may improve its healing properties. One of the most common natural organic compounds on earth is cellulose (Cel). Being biocompatible and non-toxic makes it a recommended choice for the synthesis of thin films used in wound healing [11]. Chitosan and cellulose have highly functionalized, stiff-chain, linear molecular structures but different properties that can be combined usefully. Cel primarily offers hydrophilicity, structuring, and mechanical properties, while chitosan has an electron donor function and antimicrobial properties due to the amino group present [12].

Honey (H) has an effective role in wound healing for approximately all types of wounds. It was observed that honey is preferred as a wound dressing since it can stimulate the healing process, helps clear infection, stimulates tissue regulation, and reduces inflammation [13,14]. The physical properties of honey will accelerate the healing process because the acidic pH (3.2–4.5) will promote healing by increasing the release of oxygen from hemoglobin and decreasing protease activity, thus reducing the destruction of the matrix needed for tissue repair. The high osmolarity of H due to its high sugar content improves its effectiveness as a wound dressing [15].

Curcumin (Cur), which is a natural herbal medicine that has anti-inflammatory and free-radical functions, has been proven to be a good wound-healing dressing [16]. Cur is a hydrophobic polyphenolic compound derived from the rhizome of the herb Curcuma longa. Tissue repair, anti-bacterial, anti-inflammatory, and antioxidant properties are the major properties of curcumin [17,18].

To overcome the hydrophilic and mechanical property weaknesses of CS [12], our study aims to enhance CS film properties in order to improve its effect as a dressing material by blending with Cel, H, and Cur at different concentrations [19]. Characterization properties, including X-ray diffraction (XRD), Fourier transform infrared (FTIR), and scanning electron microscopy (SEM), as well as mechanical properties and antibacterial effects, were studied.

## 2. Materials and Methods

### 2.1. Materials

Chitosan (powder with a high MW, 98% purity, DA: 93%, pH 6.5, CAS 9012-76-4, and Batch #: MKBB0585) is made in Hainan, China. The product was purchased from Giza, Egypt. Cellulose (microcrystalline, PH of suspension (2% suspension), sulfated ash, suitability for TLC) Sigma- Aldrich. Acetic Acid. Honey (100% pure honey from Imtenan Health Shop, Egypt), spring flower honey (this natural honey is acquired from sunflowers and other spring flower blossoms), and curcumin LR (curcuminoids mixture; C 1:No78300; diferuloylmethane). Natural yellow 3 (C21H20O6) MW.368.39 (Minimum assay (HPLC) 95%, melting point 175–180 °C) SDFCL was purchased from Sigma-Aldrich, Burlington, MA, USA.

### 2.2. Methods

#### 2.2.1. Sample Preparation

Seven blended films (2 films of each blend) were prepared by the casting method with different concentrations of chitosan and cellulose (CS/Cel), chitosan and honey (CS/H), and chitosan and curcumin (CS/Cur), as shown in Table 1. The film weight was dissolved in acetic acid using a magnetic stirrer and then poured into Petri dishes. All films are left at room temperature (25 °C) for a week to complete the evaporation of the solvent.

The preparation of samples was done at the research lab of the Biophysics Department, Faculty of Science, Cairo University, Giza, Egypt.

#### 2.2.2. X-ray Diffraction (XRD)

X-ray patterns for chitosan and chitosan blended with cellulose, honey, and curcumin at different concentrations were done. Powder X-ray diffraction (XRD) spectra were collected by a PANalytical diffractometer with Cu K (l = 0.154 nm) radiation, Empyrean, Netherlands. The peak positions were from 5° to 35° (2θ) with 0.013 resolution. Diffractograms were normalized to the sample amounts. These spectra were measured at the National Standard Institute, Giza, Egypt.

#### 2.2.3. Fourier Transform Infrared Spectroscopy (FTIR)

FTIR spectroscopy was carried out using a Nicolet 380 spectrophotometer (Thermo Scientific, Waltham, MA, USA). The IR spectra were scanned 64 times over the wavenumber range of 3900 to 700 cm^−1^. The seven films were measured at the National Institute of Standards (NIS), Giza, Egypt. All the spectra were acquired three times, and the program Essential FTIR was used to analyze the spectra (peak positions).

#### 2.2.4. Scanning Electron Microscopy (SEM)

Using an ion-sputtering device, the prepared films were coated with Au in order to allow the passage of electrons [20]. The conductive coating layer is responsible for the discharge of non-conductive thin films [21]. Scanning images were obtained using a Quanta FEG 250 at the National Research Centre, Giza, Egypt.

#### 2.2.5. Mechanical Properties

The mechanical properties were determined using a tensile testing machine model Z010 from Zwick Roell, Germany. Films were tested at the National Institute of Standards (NIS), Giza, Egypt. The films were cut into strip-shaped (5 cm × 5 cm) specimens. For each film, the stress and strain data were tested three times, and the average was taken. For the pure and blended films, the tensile strength, Young’s modulus, and elongation at break were measured.

T.S = the maximum force that the material withstands before breaking (F_max_/A) E-modulus or Young’s modulus = stress/strain = (F/A) (ΔL/L) (From the linear part of the stress-strain curve)

where

F is the load force
A is the cross-sectional area
ΔL is the elongation
L is the initial length

Elongation % (E %) = (ΔL/L) × 100%

#### 2.2.6. Antibacterial Effects

Gram-positive bacteria [*Staphylococcus aureus* (ATCC 6538)] and gram-negative bacteria [*Escherichia coli* (ATCC 25922)] were studied on the prepared films. The inoculation of microorganisms was prepared from fresh overnight broth cultures that were incubated at 37 °C [22]. The inoculum size of these pathogenic strains was prepared and adjusted approximately to 0.5 McFarland standard (1.5 × 10^8^ /mL), 25.0 µL of each. After the media was cooled and solidified, the prepared samples were used in the shake flask method applying nutrient broth medium (Nb).

The samples were applied on these tested microorganisms using the shake flask method to calculate the antimicrobial activity throughout (%) reduction of these microorganisms after contact with the test- samples compared to the control of the microorganisms surviving after a 24 h incubation period [23].

Reduction (%) = (A − B/A) × 100

where:

A: the number of microorganisms present in the control flask contained only bacterial strains.

B: the number of microorganisms present in the shake flask after applying the samples.

All films were tested 3 times at the National Research Centre, Giza, Egypt.

#### 2.2.7. Statistical Analysis

A 1-way analysis of variance (ANOVA) and Duncan’s test were used for data comparisons relative to pure chitosan film data using SPSS 15.0 software. Differences were considered to be statistically significant if *p* ≤ 0.05 (*). All data were presented as mean ± standard deviation (SD).

## 3. Results & Discussion

### 3.1. X-ray Diffraction (XRD)

**Figure 1:**
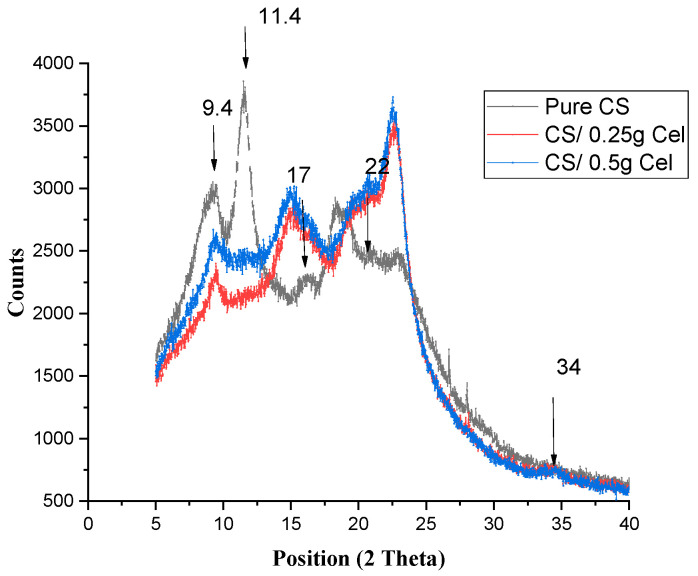
XRD for pure CS and CS-blended Cel at different concentrations.

From Figure 1, peaks of lower intensities are observed, indicating the semi-crystalline nature of pure CS film. Peaks around 2θ = 9.4°, 2θ = 11.4°, 2θ = 17°, and 2θ = 23.1° were observed. Peaks around 2θ = 9.4° and 2θ = 11.4°, which originated from (200) and (020) crystalline planes, respectively; these peaks are attributed to a hydrated crystalline structure due to the integration of water molecules in the crystal lattice. The peak around 17°originating from the (220) crystalline plane is attributed to the regular CS crystal lattice. A broad peak centered at 2θ = 22° originated from the (202) crystalline plane, indicating an amorphous structure of CS [24,25].

The broadness of the peak indicates the degree of amorphosity of the CS film. XRD patterns denote whether the blended polymers interact with each other or each one shows its own pattern [9].

For CS blended with Cel (Figure 1), the diffractogram showed the same peak of crystalline structure for CS at 9.4°, 9.5° for 0.25 Cel, and 9.4° for 0.5 Cel, while the crystallization peak disappeared at 11.4°. The peak of the regular chitosan, which is at 2θ = 17°, appeared at 14.1° and 14.9° for 0.25 g Cel and 0.5 g Cel, respectively.

The broad peak of pure CS at 2θ = 22° appeared sharper with a higher intensity at 22.6° and 22.5° in both concentrations of cellulose, respectively. The broad peak had increased in intensity while the other peaks had decreased according to pure CS. There was another small peak (hump) appearing at 34.6° and 34.5° in 0.25 g Cel and 0.5 g Cel, respectively.

The Figure showed a shift in the blended CS films at 2θ = 17°, which was shifted to a lower peak position due to Cel originating from the (101) crystalline plane, and another shift in the broad peak at 2θ = 22°, which was shifted to a higher peak position and higher intensity due to Cel originating from the (002) crystalline plane. There was another small peak (hump) appearing at 34.6° and 34.5° in 0.25 g Cel and 0.5 g Cel, respectively, originating from the (040) Cel crystalline plane [26,27].

From the XRD pattern of this blend, it was observed that blending CS with Cel decreases the semi-crystalline nature of CS due to the crosslinking between CS and cellulose. The decline in peak intensity at 9.4° at both concentrations, especially in 0.25 g Cel, can be attributed to the interferences from the co-crystallization of cellulose and chitosan, which render a less ordered structure in CS film with smaller crystallites [28,29]. This reduction in crystallinity is limited due to the crystalline nature of cellulose. The cross-linking process hindered the close packing of the polymer chains by limiting the degrees of freedom in a 3-D conformation, which limits the formation of crystalline regions [30]. Also, the original microcrystalline structure of CS induces a decrease in the film’s crystallinity [31]. Therefore, this contributes to the increase in proton transport in the amorphous phase, which leads to an increase in ionic conductivity [28]. This is more pronounced at 0.25 g Cel than at a high concentration of cellulose (0.5 g Cel).

The addition of cellulose to chitosan does not favor the crystallization of chitosan, especially at 0.25 g Cel. The broadening of the peak indicates the increase in the amorphous nature of the blend, which is due to cross-linking between CS and cellulose [1].

**Figure 2:**
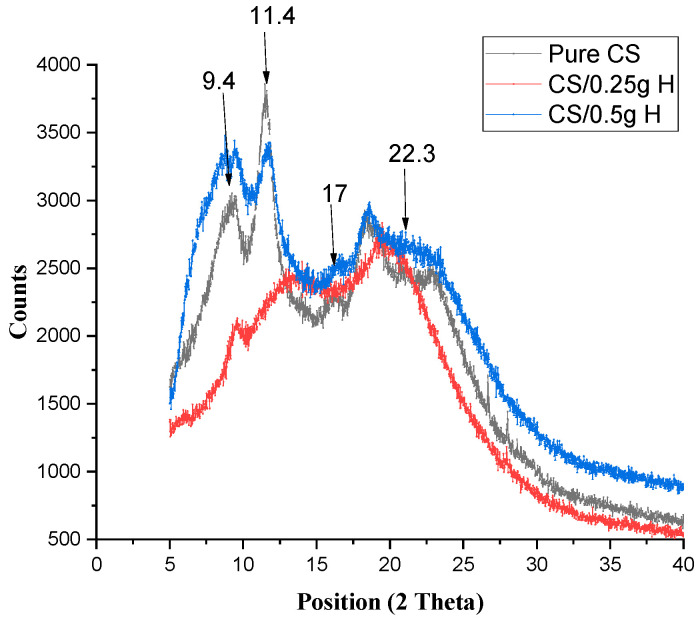
XRD for pure CS and Cs-blended H at different concentrations.

Figure 2 demonstrates the XRD of pure CS and CS/H at different concentrations. The diffractogram showed the same peak position of the crystalline structure of pure CS at 9.5° for both concentrations, with a lower intensity at 0.25 g H and a higher intensity and broadness at 0.5 g H.

The other peak of pure CS (2θ = 11.4°) was shifted to 13.0° (a broad peak with lower intensity) and 11.9° with lower intensity for 0.25 g H and 0.5 g H, respectively. The broad peak of (2θ = 22°) was shifted to the lower positions of 19.3° and 18.6° at 0.25 g H and 0.5 g H, respectively. The peak at 2θ = 17° was slightly shifted to 17.3°, appearing as a hump at 0.25 g Cur and at 17.2° at 0.5 g Cur. The broad peak at 2θ =22° appeared at 20.5° with decreasing intensity and less broadness in the case of 0.25 g Cur, while that of 0.5 g Cur appeared at 22.4° as the pure but broader peak.

Figure 2 demonstrates the hygroscopic nature of H, which increases the moisture content, which in turn increases the intensity at 0.5 g H [32].

By increasing the concentration to 0.5 g, the patterns showed a 2 shift to the left, an increase in the peak intensity, and a broadening in the peak. This is due to the formation of hydrogen bonds between H and the original microcrystalline structure of CS, resulting in decreased film crystallinity [30]. The XRD diffraction patterns of blended films exhibited a deteriorated crystalline structure compared to the pure CS film. In addition, no new peaks appear in the blended films [31].

**Figure 3:**
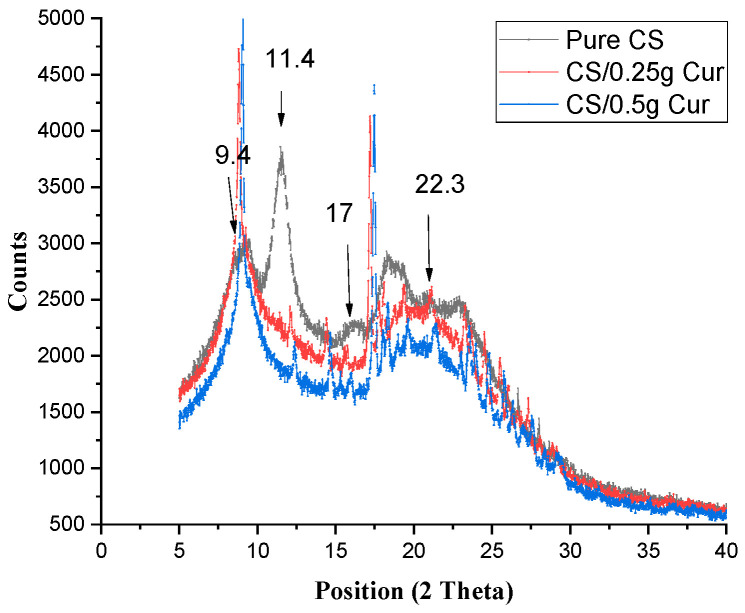
XRD for pure CS and CS-blended Cur at different concentrations.

Figure 3 shows the XRD of pure CS and CS/Cur at different concentrations. The diffractogram showed the same peak of the crystalline structure of CS at 9.4° (8.8° for 0.25 g Cur and 9.0° for 0.5 g Cur), with higher intensity and sharpness. For pure CS, 2θ = 11.4° did not appear, and there are two other peaks that appeared due to curcumin (2θ = 12.1° and 14.4° for 0.25 g Cur and 12.4°and 14.7° for 0.5 g Cur, respectively). The peak at 2θ = 17° appeared at higher positions (18.2° and 18.5° for both Cur concentrations, respectively). The broad peak (2θ = 22.4°) was slightly shifted to lower positions (21.1° and 21.5°) in both concentrations of curcumin, respectively.

There are two new peaks that appeared due to Cur (Figure 3), indicating that it appears in its crystalline form (2θ = 12.1° and 14.4° for 0.25 g Cur and 12.4° and 14.7° for 0.5 g Cur, respectively). The peak at 2θ = 17°, which was shifted to higher peak positions of 18.2° and 18.5° for both Cur concentrations, respectively, and which are more sharp, was due to curcumin’s crystalline form. The broad peak of (2θ = 22.3°) was slightly shifted to a lower peak position at 21.1° and 21.5° in both concentrations of curcumin, respectively, with decreasing intensity and broadening of the peak according to the pure CS, while the other peak increased in intensity [9,33,34].

Some peaks of Cur seem to be absent in the spectrum of the blends due to the formation of an amorphous complex with the intermolecular interaction occurring within CS and Cur molecules. This suggests a good diffusion of curcumin inside the CS matrix [33,34,35].

Cur molecules make chitosan film more flexible due to the strong interaction between CS and Cur, which destroyed the close packing of the CS matrix by forming regular crystallites, resulting in an unfavorable matrix for hydrogen bonding formation between CS chains themselves, which in turn would decrease crystallinity [5,9,33,36,37,38].

**Figure 4:**
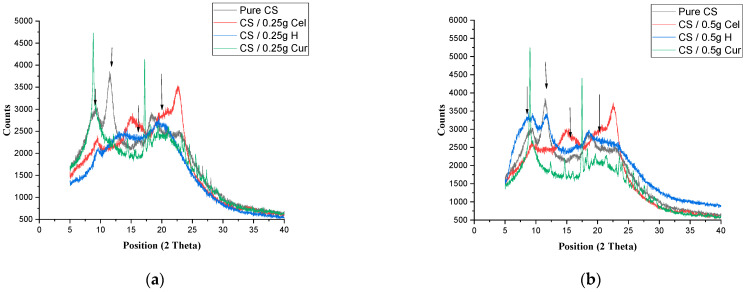
XRD for pure CS and CS-blended Cel, H and Cur for 0.25 g (**a**) and 0.5 g (**b**).

Figure 4a,b show the XRD of pure CS and CS blended with 0.25 g and 0.5 g, respectively, for the three blends. The diffractogram showed that the crystalline peak of CS at 9.4° decreases in intensity for the Cel and H blends (at 0.5 g H blend, it increases in intensity), while that of Cur tends to give a sharp and higher intensity peak. The peak of 11.4° disappears by adding the blends (in the case of 0.5 g H, it appears at a lower intensity). The peak at 2θ = 17° appeared at a lower peak position in Cel and H blends with higher intensity and more broadness, while for Cur blends, the peak is sharp and shifted to a higher position. The broad peak of (2θ = 22.4°) was slightly shifted to a lower position in 0.25 g, nearly the same as pure in 0.5 g, and had less broadness in the case of H and Cur blends, while in the case of Cel blend, it became broader with a higher peak position and intensity.

It was indicated from Figure 4a,b that the broad peak of CS at 22.3° in the case of a Cur blend has been lowered in intensity, making the peak more flat due to the interaction of phenolic compounds in Cur with the functional groups of CS. This interaction tends to have a broad peak, but it is still not as broad as the pure CS due to the crystalline nature of Cur [5].

It was also observed that this is the case in the Cel blend, but the difference is that the peak was shifted to higher positions and intensities. So, this means that there was an interaction between Cel and CS, but it was still not totally miscible. The reduction of the peak intensity means that there was a decrease in the crystallinity [39,40].

### 3.2. FTIR Spectroscopy

FTIR is a useful technique for indicating the interaction of materials with each other through shifts in wavenumbers, intensities, and disappearance of bands. This will also indicate how much the materials are compatible and miscible in their components [5].

**Figure 5:**
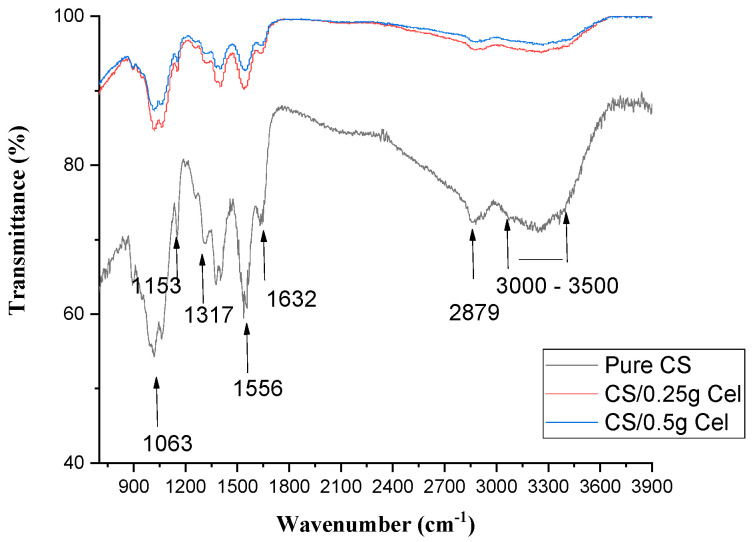
FTIR of pure CS and CS blended Cel films at different concentrations.

Figure 5 shows the FTIR of pure CS and CS blended with Cel at different concentrations. From the FTIR graphs, it is observed that the frequency of N–H bending of amide II (1556 cm^−1^) decreases with increasing Cel concentrations.

There is no change in the frequency of C=O stretching of amide I (1632 cm^−1^) by increasing Cel concentration. There were small fluctuations in the frequencies of 1317, 1153, 1063, and 1018 cm^−1^ of C–N stretching of amide III, C–O–C group, C–O stretching, and C–O vibration, respectively. C–H stretching at 2879 cm^−1^ decreases with increasing Cel concentrations.

While O–H stretching overlapping NH_2_ stretching vibration at 3000–3500 cm^−1^ increases with increasing Cel concentrations.

Figure 5 indicates the presence of good miscibility between Cel and CS due to hydrogen bond formation between their functional groups [41].

The blending of CS with Cel didn’t change the chemical structure of CS due to simple Van der Waal interactions. While chemical cross-linking between CS and Cel-containing hydroxyl groups is based on the physical interaction between the reducing ends of Cel and the primary amines of CS, promoting the dehydration of chitosan [42].

The reduction found in the N–H bending may be due to crosslinking between the CS (N–H) group and the functional group of cellulose through hydrogen bonding. With the increase in hydrogen bonding, the miscibility between the blends increases [43,44], which was also improved by the stability of C=O in amide I.

The fluctuations of C–N stretching of amide III, C–O–C group, C–O stretching, and C–O vibration may be due to H-bonding and to the carboxylic ions formed between CS and Cel [45]. The strong hydrogen bonds indicated a low vibrational frequency of the hydroxyl groups [41].

The reduction of C–H stretching is due to the formation of H-bonding between Cel and CS [44]. While O–H stretching overlaps NH_2_ stretching, vibration increases due to the interaction between the hydroxyl group or amino group of CS and the hydroxyl group of Cel. This shift increases miscibility between the blends and makes them more compatible [46].

**Figure 6:**
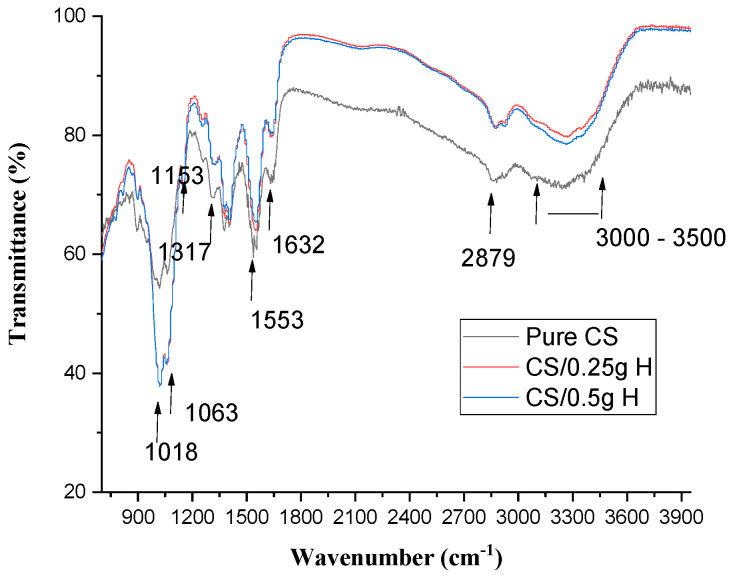
FTIR of pure CS and CS blended H films at different concentrations.

Figure 6 shows the FTIR of pure CS and CS/H at different concentrations. From the FTIR graphs, it is observed that the frequency of N–H bending of amide II (1556 cm^−1^) decreases with increasing H concentrations and then remains constant. There was a small increase in the frequency of C=O stretching of amide I (1632 cm^−1^) by increasing H concentrations.

There was an increase in the frequencies of 1317 and 1018 cm^−1^ of C–N stretching of amide III and C–O vibration, respectively. Meanwhile, there was a decrease in the frequency of 1063 and 1153 cm^−1^, corresponding to C–O stretching and C–O–C groups. There were small fluctuations in C–H stretching at 2879 cm^−1^ and O–H stretching (3000–3500 cm^−1^), which overlapped with the stretching vibration of NH_2_ by elevating H concentrations.

It was observed that C–H stretching at 2879 cm^−1^ and O–H stretching and NH_2_ vibration at 3000–3500 cm^−1^ increase in intensity in comparison to the pure and then remain constant by increasing H concentrations.

From the graph, there were no major structural changes in CS films after blending with H. In addition, there were no changes in the peak intensities except those of C–H and O–H and no major shift in the wave numbers as the weight of honey used was too small to make any shift in the chitosan spectra [47].

**Figure 7:**
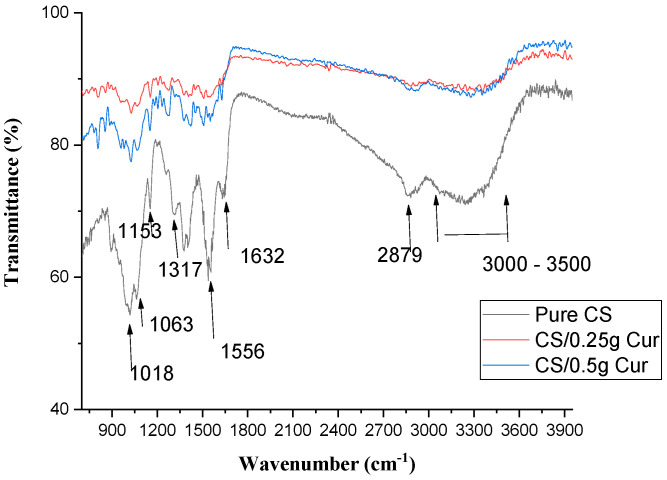
FTIR of pure CS and CS blended Cur films at different concentrations.

Figure 7 shows the FTIR of pure CS and CS/ Cur at different concentrations. From FTIR graphs, there is a decrease in wavenumber and an increase in the bond length of N–H bending amide II (1556 cm^−1^) with increasing Cur concentrations. C=O stretching of amide I and antisymmetric-NH_3_^+^ of chitosan (1632 cm^−1^) decrease with increasing Cur concentrations. Furthermore, C–H stretching at 2879 cm^−1^ and O–H stretching overlapping NH_2_ vibration at 3000–3500 cm^−1^ increase with increasing Cur concentrations.

It was observed that there was a fluctuation in the frequencies of 1317 and 1018 cm^−1^ of C–N stretching of amide III and C–O vibration, respectively. Meanwhile, there was an increase in the frequency of 1063 and 1153 cm^−1^, corresponding to C–O stretching and C–O–C groups.

The hydrogen bond interaction between CS and Cur leads to a decrease in wavenumber and an increase in bond length of N–H bending amide II by increasing Cur concentrations. The small shift in C=O stretching of amide I and the antisymmetric-NH_3_^+^ of chitosan was due to hydrogen bonds formed between the two ketone groups of curcumin and the chitosan matrix, resulting in an increased C=O bond energy [36,43]. It was observed that the height of the peaks decreases due to the interaction between Cur and CS.

The formation of H-bonding between Cur and CS leads to an increase in C–H stretching. The resulting spectra indicates the presence of a strong interaction between NH_3_^+^ of CS and OH^−^ of Cur [48]. Hence, Cur-blend films indicate that Cur and CS are completely miscible [36].

**Figure 8:**
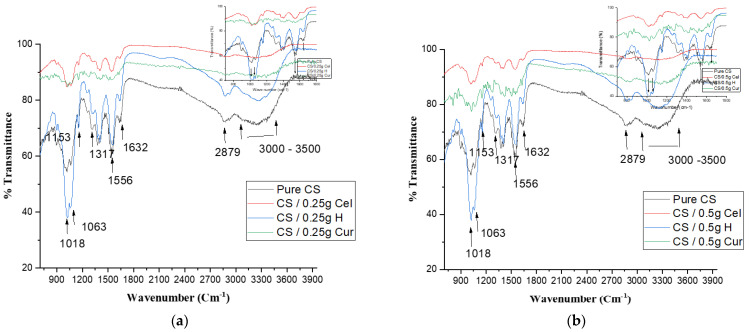
FTIR of pure CS and CS blended Cel, H, and Cur for 0.25 g (**a**) and 0.5 g (**b**).

Figure 8a,b indicate the change in CS film due to different blends by adding 0.25 g and 0.5 g, respectively. This is shown by the change in the broad OH band (3000–3500 cm^−1^), which was flatter and slightly shifted to a higher wavenumber in the Cur blend. Also, the peaks of amide I and II at 1632 cm^−1^ and 1556 cm^−1^, respectively, were flattened in the Cur blend with higher transmittance. This is due to cross-linking, which is recommended by the results of XRD.

From Figure 8a,b, the flattening of the main peak of CS of amide I and II and the broad OH peak in the case of Cur blend indicates the good miscibility between it and the CS matrix due to phenolic interaction with the CS matrix [49,50].

### 3.3. SEM

**Figure 9:**
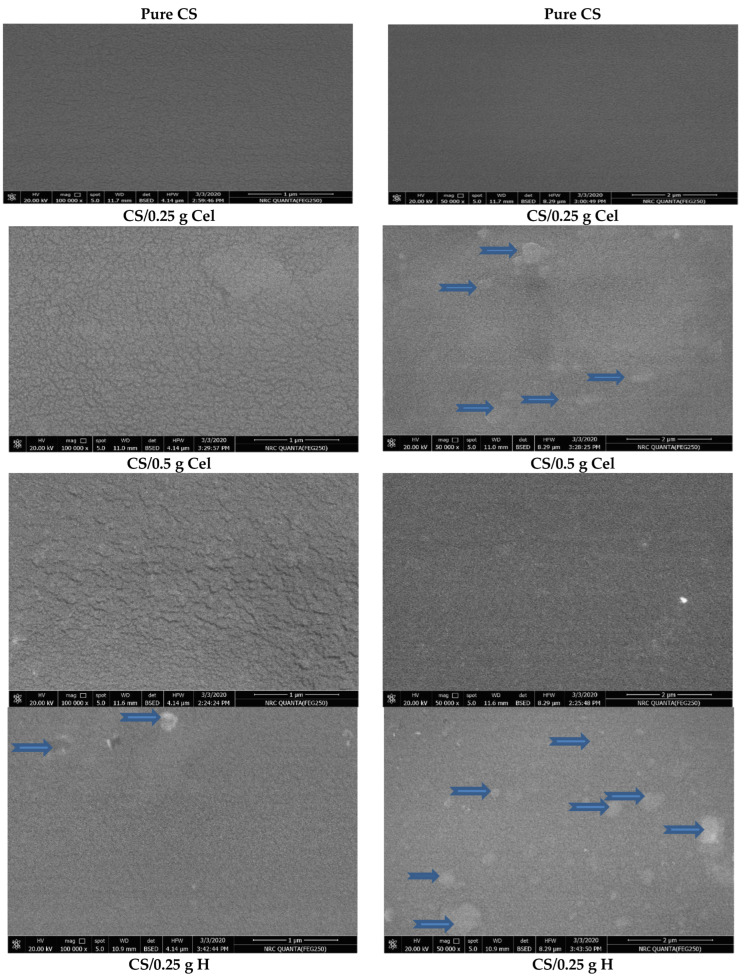

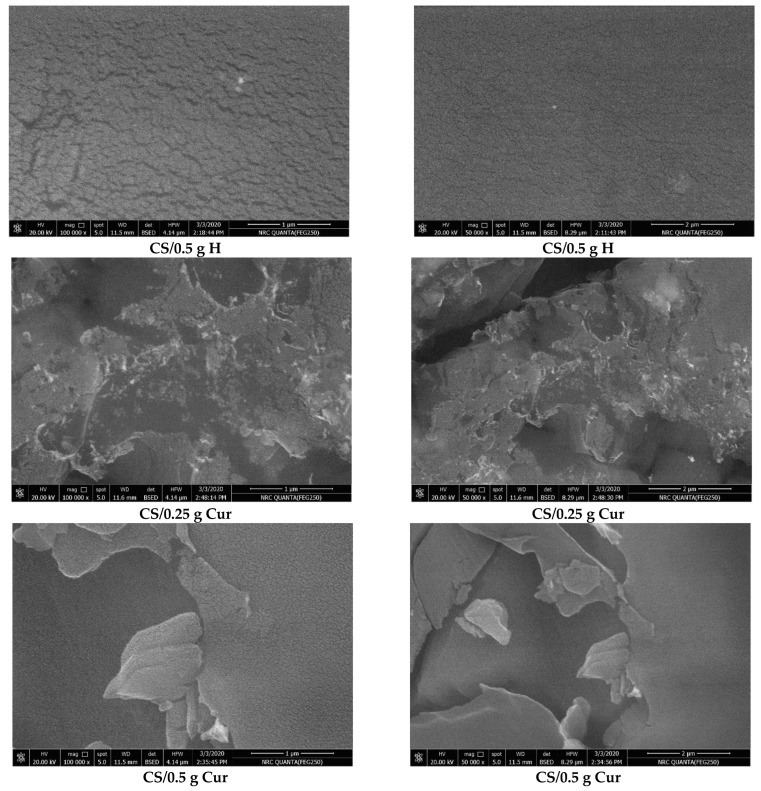
SEM for pure CS and CS blended with Cel, H and Cur at 0.25 g and 0.5 g (100,000×; to the **left**) and (50,000×; to the **right**).

Figure 9 shows SEM images of pure CS. The figure shows a relatively smooth, homogeneous, dense, and uniform planar structure without pores or cracks [5], indicating good structural integrity due to the semi-crystalline nature of chitosan [5,26,30,51,52,53].

It was observed that SEM of CS/0.25 g Cel demonstrated a rough surface with dense granules that appeared structured in lower magnification (50,000×), which makes the CS film loosely packed [28,51,54,55]. Also, CS/0.5 g Cel showed more rough and non-uniform structures with fewer granules. The granules found in 0.25 g and 0.5 g of Cel may be attributed to strong molecular inter-atomic interactions between Cel and CS. This correlates well with the changes in crystallinity, indicating loosely packed microstructures aligned with the highly hydrophobic surface of the CS matrix. Blending cellulose with CS improved the porosity of CS film [26,28,51,53,54].

SEM of CS/HH showed notable nodes at 0.25 g H. While at high concentrations of 0.5 g H, the number of nodes decreases, and a compact surface with good structural integrity is observed, especially at 50,000× [56,57].

The increase in the number of pores at 0.25 g H is an advantage for tissue engineering and wound healing applications [32].

The addition of honey, especially at 0.25 g H, stimulated the physical interaction in the CS matrix by diminishing chain interactions (lower entanglement and/or crystallites), and this is in good agreement with XRD results [57,58,59]. By increasing the chitosan concentration in the blended films (0.25 g H), the attraction between the anion and cation decreases. The cations from CS may form intramolecular bonds rather than intermolecular bonding with H due to the lack of anion in honey’s molecular structure. Intramolecular bonding led to a reduction in film rigidity [60].

By increasing the concentration of H (0.5 g), the interaction between CS and H occurs, which will increase the cross-linking. The compact surface of 0.5 g H means that there is good interfacial adhesion between CS and H due to the stickiness of honey [61]. This is demonstrated in the results of the XRD.

From the figure, it was observed that the SEM images of CS and Cur with different concentrations exhibited a dense, spongy-like cross-section morphology with a disconnected structure. The spongy structures of CS blended with Cur are due to the strong interaction between CS and Cur, which causes a disruption in the morphology of CS films [62,63]. This finding is consistent with the results of XRD.

### 3.4. Mechanical Properties

**Figure 10:**
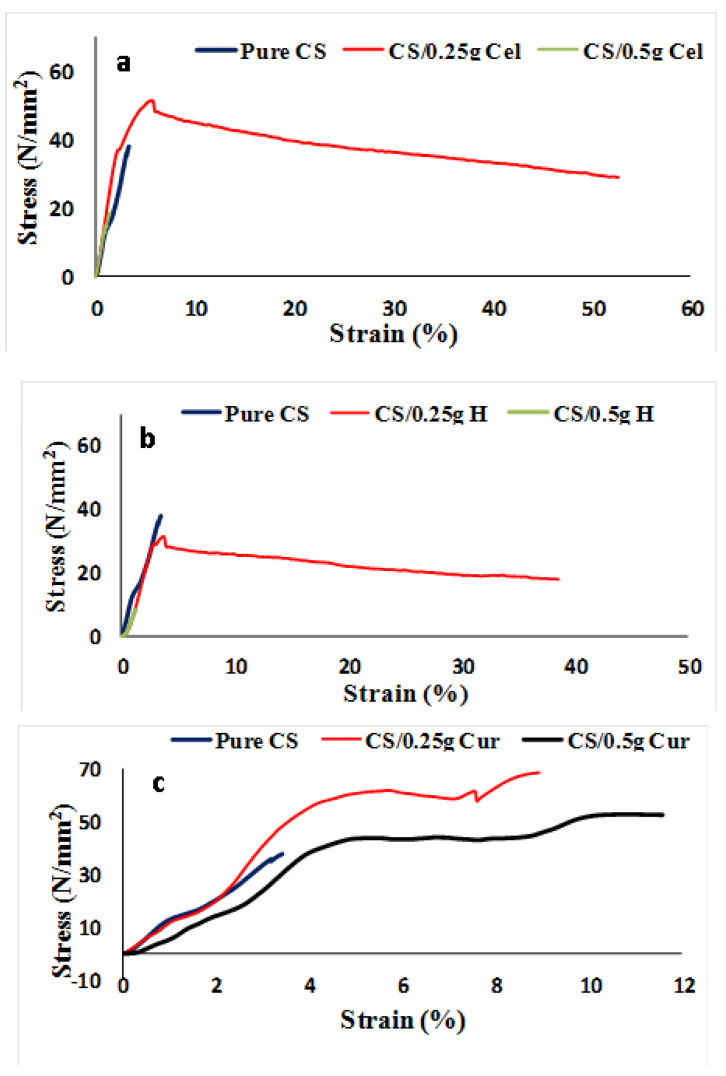
Stress-strain curves for pure CS and CS blended films (**a**–**c**) at different concentrations.

The stress-strain curves for pure chitosan (CS) and chitosan blended with cellulose, honey, and curcumin at different concentrations are shown in Figure 10.

**Figure 11:**
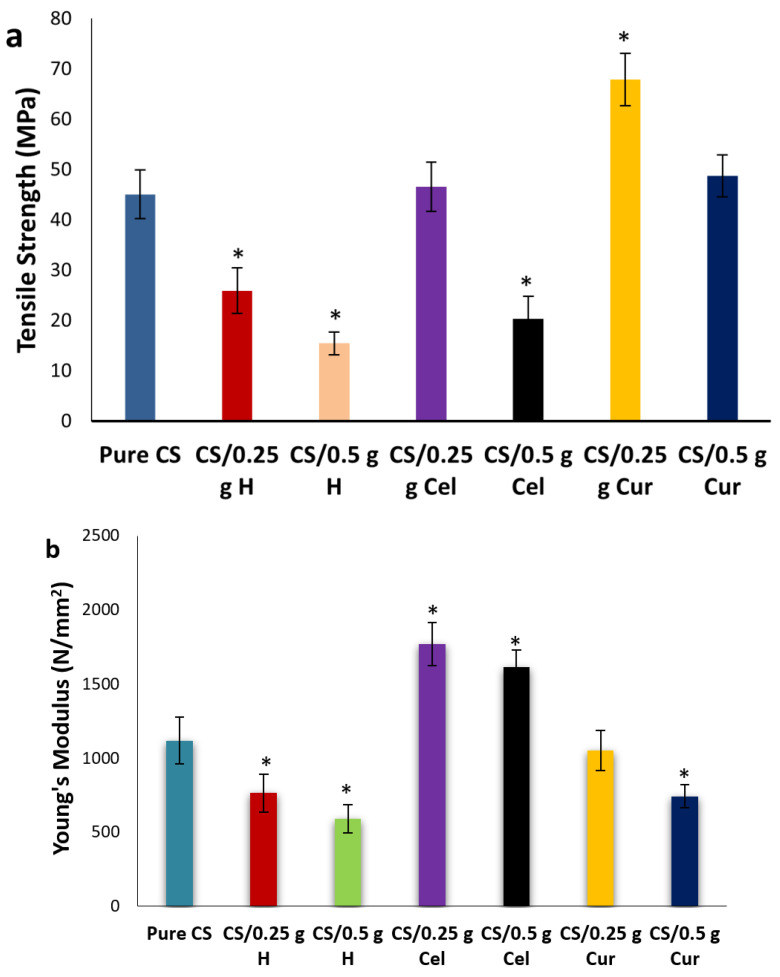

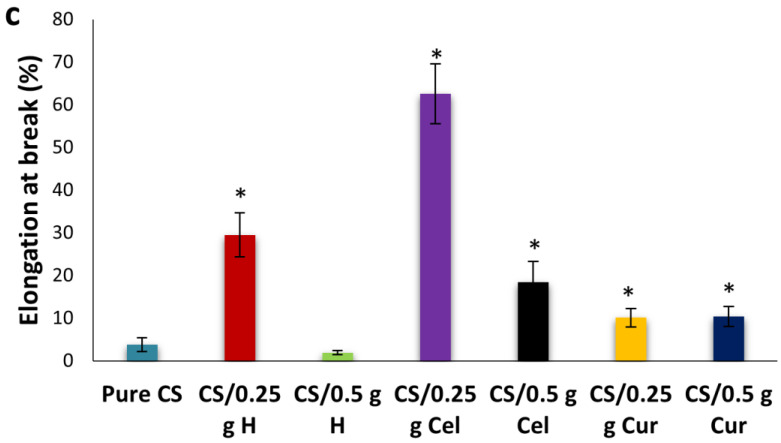
Tensile strength (**a**), Young’s modulus (**b**) and % elongation at break (**c**) for pure CS and CS-blended samples at different concentrations [* means that there is a significant change (*p* ≤ 0.05)].

From stress-strain curves (Figure 10), the tensile strength, Young’s modulus, and elongation at break for pure CS, CS/Cel, CS/HH and CS/Cur at different concentrations were calculated and presented in Figure 11. Pure CS film shows low tensile strength, which is due to its low water resistance properties, and this gives chitosan its poor mechanical properties [64,65,66,67]. In order to improve these properties, CS films were blended with natural materials because chitosan films with higher tensile strength and reasonable elongation at break values have a higher potential in medical and other applications [68,69].

Tensile strength, Young’s modulus, and percentage elongation at break show an increase and a decrease at 0.25 g Cel and 0.5 g Cel, respectively. Young’s modulus at 0.5 g Cel was decreased in comparison to 0.25 g Cel but still higher than that of pure CS.

The increase in tensile strength and elongation at break at 0.25 g Cel indicates that there are intermolecular interactions between the -OH group of cellulose and the -OH and -NH_2_ groups of chitosan, which leads to a decrease in crystallinity. This will restrict the motion of the matrix while promoting rigidity [70,71,72,73]. This is in agreement with FTIR graphs, which show that by increasing the absorption peak frequency of O-H and C-H bands, the elongation at break will increase [46].

Blending with Cel increases the water resistance and the thermal stability of CS films, and this will increase the tensile strength and the elongation at break [51]. The addition of cellulose shows an enhancement of the mechanical properties to a certain level. Increasing the concentration of Cel has reduced the plasticity of CS films and improved the stiffness of the blend films [74].

The decrease in crystallinity means that the amorphous region has been enlarged, which leads to increased conductivity. This is in good agreement with the XRD results.

The significant reduction (*p* ≤ 0.05) of tensile strength and elongation at break at 0.5 g Cel was due to the intramolecular bonds rather than the intermolecular bonds between chitosan and cellulose. Cel at 0.5 g didn’t have homogeneous interaction with CS at 0.25 g, leading to a reduction of the mechanical properties by decreasing the tensile strength and increasing the probability of fracture [44,75,76,77].

The reduction in elongation at break may be attributed to the stiffening effect of the highly crystalline cellulose, which increased the rigidity of the blend. Weak adhesion between CS and Cel interfaces causes a decrease in the affinity of the CS matrix. Therefore, 0.5 g Cel blended films will become more flexible and less compact [76].

For CS-blended H, CS/0.25 g H thin films showed a significant decrease in tensile strength and Young’s modulus and a significant increase in elongation at break (*p* ≤ 0.05). The reduction in strength and the increment of elongation at the break by adding H may be due to the reduction of hydrogen bonds due to the intramolecular bonding of CS itself rather than intermolecular bonding with H and the increment of thickness and weight [78,79,80]. By increasing the concentration of H to 0.5 g, the tensile strength and percent elongation were decreased.

Intramolecular bonding led to a reduction in film rigidity and decreased tensile strength [60]. Also, lower tensile strength and Young’s modulus may be due to the increased porosity of the films [47,60,68]. This was confirmed by the SEM.

For CS-blended Cur, the tensile strength and elongation at break for 0.25 g Cur increased significantly (*p* ≤ 0.05), while Young‘s modulus decreased [20,81].

The increment of tensile strength at 0.25 g Cur means that more energy is required to break the films due to the hydrogen bond network between chitosan and curcumin [80,81].

The tensile strength of 0.5 g of Cur is high in comparison to that of pure CS but lower than that of 0.25 g. Also, elongation at break significantly increased while Young’s modulus significantly decreased (*p* ≤ 0.05). The increase in tensile strength and elongation at break means more strength and flexibility in the films. Due to the intermolecular interaction between -OH and -NH_2_ groups between CS and Cur, the blending improves the mechanical properties of chitosan films [19,33,82]. In addition, the highest tensile strength may be due to the electrostatic bonding of the -NH_2_ and -NH_3_ groups in the CS chains [83]. This finding is compatible with the aforesaid results of XRD and FTIR [5].

The induction of a local stress concentration of Cur in the CS matrix at 0.5 g Cur leads to a reduction in tensile strength and Young‘s modulus [83].

The addition improved tensile strength. The cyclic chain in CS molecules can aid in bonding the functional groups (OH and NH_2_) with other molecules. The H-bonds formed were responsible for the mechanical properties of CS by inhibiting the rotation of chain molecules due to cross-linking. By adding Cur, the molecular chain rotation and the tensile strength have been increased due to high-energy intermolecular interactions in which Cur acts as the CS molecules’ interface coupling agents [5]. The cross-linking between CS and Cur improves the tensile strength and decreases the elasticity [37,82]. This will make CS-blend Cur films more rigid than pure CS films [33,37,82]. This is confirmed by the results of the XRD and SEM.

**Figure 12:**
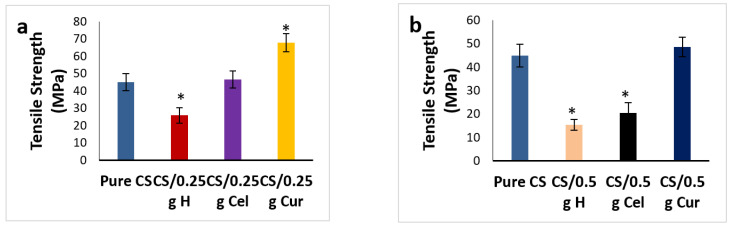
Tensile strength for pure CS and CS-blended samples at 0.25 g (**a**) and 0.5 g (**b**) [* means that there is a significant change (*p* ≤ 0.05)].

Figure 12a,b show the effect of different blends at 0.25 g and 0.5 g, respectively, on the tensile strength. The tensile strength was significantly enhanced in the case of the Cur blend, then decreased in the Cel blend and H blend (*p* ≤ 0.05). This indicates that there was good miscibility in the case of Cur rather than Cel and H.

**Figure 13:**
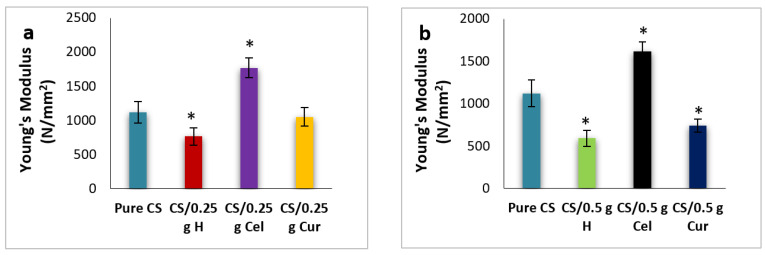
Young’s modulus for pure CS and CS-blended samples at 0.25 g (**a**) and 0.5 g (**b**) [* means that there is a significant change (*p* ≤ 0.05)].

Figure 13a,b show the effect of different blends at 0.25 g and 0.5 g, respectively, on the Young’s modulus. There was a significant decrease in modulus (*p* ≤ 0.05) in cases of H and Cur blends in comparison to the pure. The contrary was observed in the case of Cel. The reduction in modulus makes the film more elastic.

**Figure 14:**
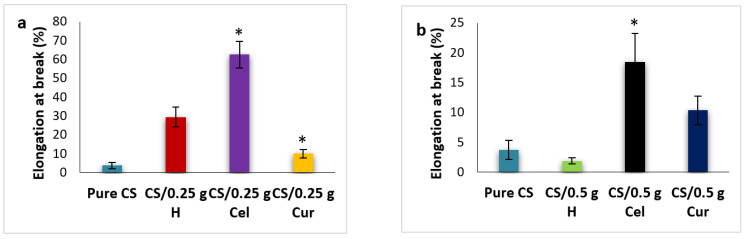
Percent elongation at break for pure CS and CS-blended samples at 0.25 g (**a**) and 0.5 g (**b**) [* means that there is a significant change (*p* ≤ 0.05)].

Figure 14a,b show the effect of different blends at 0.25 g and 0.5 g, respectively, on the elongation at break. The elongation at 0.25 g has been significantly increased in the case of Cel, while it decreases significantly (*p* ≤ 0.05) in the case of H and decreases too much in the case of Cur, but is still higher than the pure. While at 0.5 g, the contrary is true: the elongation decreases in Cel and H compared to the pure and increases in Cur. The increase in elongation is due to intramolecular hydrogen bonding rather than interatomic hydrogen bonding. This will lead to a reduction in film rigidity [44,60]. Cur-blended CS films were more elastic and rigid than other blended CS films.

### 3.5. Antibacterial Effect

The antibacterial mechanism of CS was mainly due to the effective NH_2_ group. The protonated NH_3_^+^ groups can combine with the electronegative charges of biomolecules on the microbial bacterial surface to change the permeability of the membrane and induce the leakage of proteinaceous and other intracellular constituents to inhibit the growth of the bacteria. It was observed that as the molecular weight of CS increased, the antibacterial effect against gram-positive bacteria increased but decreased against gram-negative bacteria [30,44,59].

The behavior of pure CS against gram-negative and gram-positive bacteria may be due to the acidic conditions of the film that cause CS to be protonated. The positive charge of CS decreases the osmotic stability of the bacterial cell membrane. This will alter cell permeability and reduce intracellular contents, resulting in cell rupture. In addition, positively charged chitosan may interact with the negatively charged teichoic acids in the cell wall of gram-positive microbes, leading to the formation of small pores on the cell wall and subsequently leading to leakage of the intracellular components [42]. Also, the low molecular weight of chitosan affects its activity.

Table 2 shows that pure CS has a mild effect against *E. coli* (gram-negative bacteria) and no effect against *Staphylococcus aureus* (gram-positive bacteria). Bacterial cell lysis due to the release of cellular contents is the reason for the mild effect (Nil, meaning no cell reduction or very small cell reduction) of pure CS against *E. coli*. This is due to the increment of the bacterial outer membrane permeability [25,47].

It was observed that CS and Cel films exhibited a strong and significant antimicrobial effect (*p* ≤ 0.05) against *E. coli and S. aureus,* especially at 0.25 g. So, CS/Cel blend films can be used as biomaterials, medical dressings, and skin tissue engineering [44]. The strong antimicrobial effect may be due to the strong interaction between cellulose and chitosan.

For CS-blended H, 0.25 g H concentration shows a significant (*p* ≤ 0.05) antibacterial effect against gram-negative bacteria, while a small significant antibacterial effect was realized at 0.5 g H. No antibacterial effect was observed against *S. aureus* at 0.5 g H [32].

The antibacterial effect of H comes from its acidity, high sugar content, and hydrogen peroxide production ability. On the other hand, the polycationic nature of CS allows it to interact with the bacterial cell membrane, resulting in reduced membrane permeability with subsequent cell leakage and death [32].

Based on the amount of sugar that affects the activity against *S. aureus* (bacteriostatic or bactericidal), it was observed that 0.25 g H usually presented activity against *S. aureus*, while 0.5 g did not, probably due to the non-peroxide components. Honey is also usually used as a co-adjuvant to antibiotics in treatment against *S. aureus*, although the effectiveness of honey is dependent on its concentration [56,58].

From the table, it was observed that the antibacterial effect for *E. coli* decreases at 0.25 g Cur and then significantly increases at 0.5 g (*p* ≤ 0.05). In contrast to *S. aureus*, it significantly increases sharply at 0.25 g while disappearing at 0.5 g.

From Table 3, it is observed that the significant reduction of bacteria, especially *E. coli*, increases in the Cel blend, followed by the H blend, and decreases in the Cur blend in comparison to the pure. While in *S. aureus*, CS-blended Cur demonstrated the highest significant reduction (*p* ≤ 0.05), followed by CS-blended Cel.

At high concentrations of the blends (0.5 g; Table 4), in the case of *E. coli*, the Cur blend showed the highest significant reduction, followed by the Cel blend. While at *S. aureus*, the Cel blend only showed a significant reduction, and there was no reduction in the other blends.

Therefore, Cur can improve the antibacterial effect against *S. aureus* at low concentrations due to intermolecular interactions by keto-enol tautomerism. One hydrogen bond is formed by OH, and the other is formed by NH_2_ of the glucosamine of CS to the oxygen of OH on the benzene ring of Cur. This will inhibit the growth of these gram-negative bacteria [33].

From the results, it was observed that the Cur blend at 0.25 g had the highest antibacterial effect against *S. aureus* and that at 0.5 g had the highest effect against *E. coli*. This is due to the cross-linking of Cur with CS chains, which leads to the suppression of the microorganism’s cell wall. After entering the microorganism cell, they inhibit nucleic acid synthesis, leading to protein formulation inhibition and disruption in metabolism activities [5]. This was improved by the results of XRD, SEM, and mechanical.

## 4. Conclusions

In this study, CS films were blended with Cel, H, and Cur at different concentrations. Mechanical properties, XRD, FTIR, SEM, and antibacterial effects were investigated. XRD and mechanical results confirmed that Cur crosslinks with CS chains, resulting in more rigid and amorphous films (especially at 0.25 g). The addition of curcumin increases tensile strength and rigidity, which will allow the film to be used as a wound dressing. While other blended films had a small effect as Cel (which showed an increase in stiffness but less than that blended with Cur) or no effect as H. So, Cur-blended CS films had a high potential for use as wound dressings [84].

## Figures and Tables

**Table 1:** Representation of the weight of CS/Cel, CS/H and CS/Cur blended films.

CS Wt. (g)	Blend Wt. (Cel, H, Cur) (g)	Acetic Acid Volume (mL)	Distilled Water Volume (mL)
2	0	2	98
1.75	0.25	1.75	98.25
1.5	0.5	1.5	98.5

**Table 2:** The reduction (%) of bacterial strain cells after 24 h incubation of applying samples [* means that there is a significant change (*p* ≤ 0.05)].

Samples	Pure CS	CS/0.25 g Cel	CS/0.5 g Cel	CS/0.25 g H	CS/0.5 g H	CS/0.25 g Cur	CS/0.5 g Cur
*Escherichia coli*	27.64	55.2 *	29.62	49.3 *	4.43 *	23.7	36.51 *
*Staphylococcus aureus*	NiL	32.1 *	29.24 *	28.7 *	NiL	50.4 *	NiL

**Table 3:** The reduction (%) of bacterial strain cells after 24 h incubation for 0.25 g CS blends [* means that there is a significant change (*p* ≤ 0.05)].

Samples	Pure CS	CS/0.25 g Cel	CS/0.25 g H	CS/0.25 g Cur
*Escherichia coli*	27.64	55.2 *	49.3 *	23.7
*Staphylococcus aureus*	NiL	32.1 *	28.7 *	50.4 *

**Table 4:** The reduction (%) of bacterial strain cells after 24 h incubation for 0.5 g CS blends [* means that there is a significant change (*p* ≤ 0.05)].

Samples	Pure CS	CS/0.5 g Cel	CS/0.5 g H	CS/0.5 g Cur
*Escherichia coli*	27.64	29.62	4.43 *	36.51 *
*Staphylococcus aureus*	NiL	29.24 *	NiL	NiL

## Data Availability

Not applicable.
